# [^177^Lu]Lu-PSMA radioligand therapy as an initial approach in hormone-sensitive metastatic prostate cancer: a case report

**DOI:** 10.1007/s00508-025-02642-3

**Published:** 2025-11-06

**Authors:** Ilva Kristiana Langrate, Elisabeth Kretschmer-Chott, Stefan Schmitl, Shahrokh F. Shariat, Marcus Hacker, Gero Kramer, Sazan Rasul

**Affiliations:** 1 Department of Biomedical Imaging and Image-Guided Therapy, Division of Nuclear Medicine, Medical University of Vienna, Vienna, Austria; 2 Department of Urology, Comprehensive Cancer Center, Medical University of Vienna, Vienna, Austria; 3 Department of Urology, University of Texas Southwestern Medical Center, Dallas, USA; 4 Division of Urology, Department of Special Surgery, The University of Jordan, Amman, Jordan; 5 Department of Urology, Second Faculty of Medicine, Charles University, Prague, Czech Republic; 6 Department of Urology, Weill Cornell Medical College, New York, USA; 7 Karl Landsteiner Institute of Urology and Andrology, Vienna, Austria

**Keywords:** PSMA radioligand therapy, Androgen deprivation therapy, mHSPC, PSA, Theranostics

## Abstract

The [^177^Lu]Lu-PSMA radioligand therapy is a prostate-specific membrane antigen (PSMA) targeting treatment approved for patients with metastatic castration-resistant prostate cancer, after failure of androgen deprivation therapy or taxane-based chemotherapy. We report a unique case of a patient with metastatic hormone-sensitive prostate cancer (mHSPC) who received PSMA radioligand therapy as the sole treatment, without any prior or additional systemic treatment. Over a span of 8 years, the patient underwent 12 cycles of [^177^Lu]Lu-PSMA radioligand therapy, demonstrating favorable therapeutic response and a prolonged androgen deprivation therapy-free period. This case highlights the potential of long-term PSMA radioligand therapy as a standalone option in selected mHSPC patients.

## Case report

Radioligand therapy (RLT) using ^177^Lu-labelled compounds is an approved therapy for patients with prostate-specific membrane antigen (PSMA) positive metastatic castration-resistant prostate cancer (mCRPC) [1]. The PSMA is overexpressed in prostate cancer (PCa) cells, making it a promising target for individualized treatment. After binding, the ^177^Lu-labelled compound incorporates into the cell, resulting in β‑ particle emission and PCa cell death [2].

The role of PSMA-RLT in metastatic hormone-sensitive prostate cancer (mHSPC) remains uncertain. The UpFrontPSMA trial demonstrated that [^177^Lu]Lu-PSMA-RLT prior to chemotherapy improved the PSA response and clinical outcomes versus chemotherapy alone in patients with mHSPC [3]. Here, we report on a patient with mHSPC who bypassed conventional therapies and opted for [^177^Lu]Lu-PSMA RLT as the initial treatment.

A 61-year-old male was diagnosed with PCa in 2010, with an initial prostate-specific antigen level of 74.1 μg/l. A biopsy confirmed a prostate adenocarcinoma with a Gleason score 7 (4 + 3). Following radical prostatectomy, histopathology revealed a Gleason score of 9 (4 + 5), staged as pT3b, with 1/6 obturator lymph nodes positive (pN1). Imaging revealed metastasis in local lymph nodes, prompting initiation of androgen deprivation therapy (ADT) that decreased prostate specific antigen (PSA) to a nadir of 0.1 ng/ml. The ADT was discontinued after 3 months due to intolerable side effects, including fatigue, hot flushes and musculoskeletal pain. Despite discontinuation, testosterone remained at castration level until late 2012.

In March 2016 the PSA reached 16 μg/l (PSA doubling time: 7 months), indicating a high-risk biochemical recurrence. Positron emission tomography with magnetic resonance imaging (PET/MRI) revealed local recurrence with PSMA-avid lymph nodes in parailiacal, presacral and pararectal regions. The PET/MRI was performed on a Biograph mMR system (Siemens, Erlangen, Germany) 60 min after 2 MBq/kg [^68^Ga]Ga-PSMA-11 injection; a skull base to thigh scan was acquired in 4 bed positions of 5‑min each with standard acquisition parameters. At a multidisciplinary oncology meeting, PSMA-RLT was recommended over standard options, given the imaging findings, PSA increase, ADT intolerance and patient preference.

**Fig. 1:**
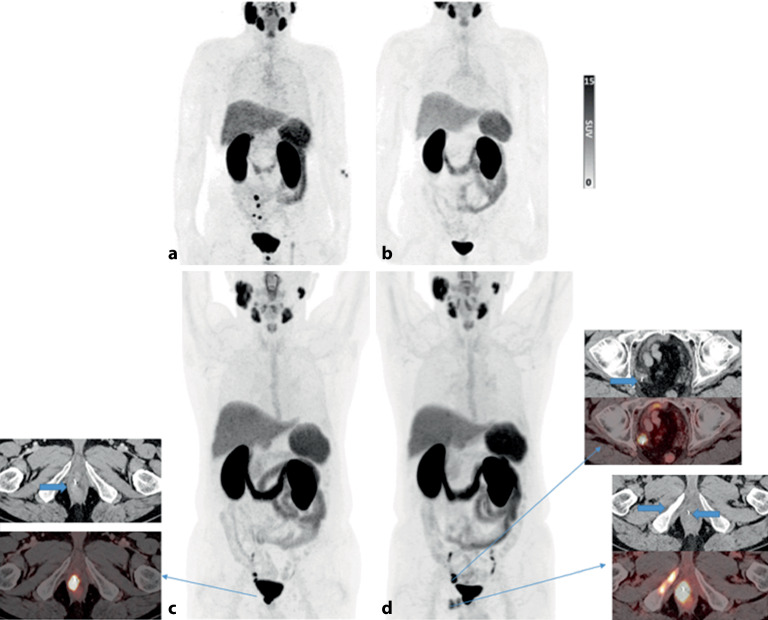
[^68^Ga]Ga-PSMA-11 PET skull base to thigh scans throughout multiple cycles of [^177^Lu]Lu-PSMA RLT. **a** [^68^Ga]Ga-PSMA-11 PET imaging demonstrates PSMA-avid lymph node metastases and local recurrence after radical prostatectomy in the pelvic region before therapy initiated in March 2016; **b** PSMA-PET showing a reduction in volume and avidity of multiple PSMA lymph nodes in the retroperitoneal and right parailiac regions after three PSMA-RLT cycles in August 2016; **c** [^68^Ga]Ga-PSMA-11 PET/CT imaging before the second rechallenge with progressive local recurrence and enhanced PSMA uptake in retroperitoneal lymph nodes (blue arrows); **d** PSMA-PET/CT after completion of 12 RLT cycles demonstrates progression in right parailiacal lymph nodes, further progression of local recurrence in prostate bed, and a new lesion in the right pubic bone, marked by blue arrows.

From March to June 2016, the patient received first three cycles of [^177^Lu]Lu-PSMA-RLT (average 7630 MBq per cycle), resulting in 98% PSA reduction (from 16.0 μg/l to 0.17 μg/l) and markedly reduced PSMA-expression in lymph nodes and the prostate region (Fig. 1). Each cycle was accompanied by antiemetic prophylaxis and nephroprotective hydration. No therapy-related side effects were reported. Given the favorable response, further therapy was withheld. The PSA and PET monitoring continued every 3–5 months, with the patient remaining asymptomatic.

In March 2021 the PSA rose to 9.67 μg/l and 13.7 μg/l by August, with PSMA positron emission tomography with computer tomography (PET/CT) revealing PSMA-avid lymph nodes in the right paracaval and parailiac regions. The PET/CT was performed 60 min after injection of 2 MBq/kg [^68^Ga]Ga-PSMA-11 on a Siemens Biograph TruePoint, with standard PET and CT acquisition settings. Based on the findings, a PSMA-RLT rechallenge was initiated with six RLT cycles (October 2021–May 2022; average 7430 MBq per cycle), resulting in an 80% PSA decline (from 14 μg/l to 2.8 μg/l) and regression of local recurrence and PSMA uptake in the paracaval, parailiac and obturator lymph nodes. Given the significant PSA decline and radiographic findings, therapy was paused with the patient remaining on active surveillance. Regular monitoring demonstrated sustained disease control with minimal toxicity throughout multiple cycles of RLT (Table 1).

**Table 1:** Overview of hematological parameters throughout the RLT

Date	Hemoglobin (14–18.0 g/dL)	Leukocytes (4.0–10.0 10^9^/L)	Platelets (150–350 10^9^/L)	Creatinine (0.70–1.20 mg/dL)	Alkaline phosphatase (40–130 U/L)	Lactate dehydrogenase (< 250 U/L)
December 2015	17.2	7.45	223	0.91	*39*	165
December 2016	17.2	6.9	184	0.87	*34*	160
January 2018	17.3	7.38	197	0.84	*34*	168
September 2019	17.2	6.77	194	0.9	*38*	196
August 2020	16.9	6.1	201	0.86	*36*	168
October 2021	16.6	5.97	188	0.87	44	166
June 2023	15.9	5.97	198	1.08	52	198
October 2024	14.2	7.97	225	*1.26*	77	*257*

Hematological and biochemical parameters monitored throughout the 8‑year course of RLT. All values remained largely within the normal ranges given in the headings, indicating preserved bone marrow and renal function, with only mild fluctuations observed over time; numbers shown in italic are outside the normal range

In February 2023 the PSMA PET/CT revealed new PSMA-expressing lymph nodes in the para-aortic and obturator regions. By February 2024 the patient’s PSA increased to 41.6 μg/l, prompting an additional three cycles of PSMA-RLT (average 7513 MBq per cycle), after which PSA declined to 24.0 μg/l in May 2024. The [^68^Ga]Ga-PSMA-11 PET/CT showed reduction of PSMA-expressing local recurrence and a new PSMA-expressing osseous lesion in the inferior pubic ramus (Fig. 1).

In December 2024, reaching a PSA level of 79.3 μg/l, the patient began systemic ADT. By May 2025, a fast and deep PSA response was achieved, with PSA level declining to 0.04 μg/l, indicating hormone-sensitive PCa.

## Discussion

Treatment options for patients with mHSPC have greatly evolved since 2015, when ADT alone was the standard of care, docetaxel was reserved for high-volume disease, while androgen receptor pathway inhibitors (ARPIs) were only beginning to emerge [4]. By 2025, treatment intensification with long-term ADT combined with docetaxel and ARPI had been shown to prolong overall survival (OS), radiographic progression-free survival and delayed progression to CRPC [5]. Therefore, if the same patient were treated in 2025, triplet therapy (ADT + ARPI + docetaxel) or doublet therapy (ARPI + ADT) would have been highly recommended. Nevertheless, in our case, the patient achieved excellent results with RLT alone, reaching progression-free survival of 38 months, an ADT therapy-free survival of 104.9 months and OS without long-term toxicity, while remaining hormone-sensitive.

As shown in various studies, long-term ADT increases the risk of cardiovascular diseases, diabetes and osteoporosis [6], prompting further investigation into the use of RLT in earlier disease settings. In a single-center study, Sathekge et al. evaluated ^225^Ac-PSMA-617 as an initial treatment for 21 treatment-naive mHSPC patients, reporting a PSA decline ≥ 50% of the baseline in 86% (18/21) of patients and a favorable safety profile, with grade I/II xerostomia being the most common adverse effect (94%) [7]. The use of PSMA-RLT has been associated with less severe side effects, including myelotoxicity, xerostomia and fatigue [8]. A potential long-term side effect of PSMA-RLT is nephrotoxicity due to PSMA expression on renal cells and excretion of [^177^Lu]Lu-PSMA [9]. Here, the patient reported no therapy-associated side effects and laboratory findings showed no signs of nephrotoxicity.

Given the significant heterogeneity of mHSPC, selecting the most beneficial treatment approach should be guided by multiple prognostic indicators, rather than relying solely on the Eastern Cooperative Oncology Group (ECOG) status and disease volume. Additional consideration should be given to clinical factors (e.g., age and comorbidities), analytical parameters (hematological findings), pathological characteristics (Gleason score, genetic variations) and imaging findings to enable a personalized approach that enhances patient outcome [10]. Positive predictive factors for initiating RLT include having lymph node metastasis only, ECOG < 2 and no prior chemotherapy [11], while an early PSA decline ≥ 30% after 2 RLT cycles can help identify patients likely to benefit from further RLT or a rechallenge PSMA-RLT [12]. Poor PSMA-RLT outcomes are associated with symptomatic disease, high Gleason score, visceral/bone metastases, elevated lactate dehydrogenase, alkaline phosphatase and low hemoglobin levels [13]. In this case report, we present a chemotherapy-naïve, asymptomatic mHSPC patient with ECOG 0, nodal lesions on PSMA-PET imaging and laboratory values within normal ranges, who showed a PSA decline following the initial two cycles of PSMA-RLT indicating a response to treatment. These favorable baseline factors and treatment outcomes suggest that with close monitoring, long-term PSMA-RLT can offer an alternative for well-selected mHSPC patients to prolong ADT therapy-free survival and delay castration resistance.

## References

[1] Sartor O, de Bono J, Chi KN, et al. Lutetium-177–PSMA-617 for metastatic castration-resistant prostate cancer. N Engl J Med. 2021;385:1091–103. 34161051 10.1056/NEJMoa2107322 PMC8446332

[2] Kouri MA, Georgopoulos A, Manios GE, et al. Preliminary study on lutetium-177 and gold nanoparticles: apoptosis and radiation enhancement in hepatic cancer cell line. CIMB. 2024;46(11):12244–59. 39590321 10.3390/cimb46110727 PMC11592690

[3] Azad AA, Bressel M, Tan TH, et al. Sequential [^177^Lu]Lu-PSMA-617 and docetaxel versus docetaxel in patients with metastatic hormone-sensitive prostate cancer (upfrontPSMA): a multicenter, open-label, randomised, phase 2 study. Lancet Oncol. 2024;25(10):1267–76. 39293461 10.1016/S1470-2045(24)00440-6

[4] Ng K, Smith S, Shamash J. Metastatic hormone-sensitive prostate cancer (mHSPC): advances and treatment strategies in the first-line setting. Oncol Ther. 2020;8(2):209–30. 32700045 10.1007/s40487-020-00119-z PMC7683690

[5] Fizazi K, Foulon S, Carles J, et al. Abiraterone plus prednisone added to androgen deprivation therapy and docetaxel in de novo metastatic castration-sensitive prostate cancer (PEACE-1): a multicentre, open-label, randomised, phase 3 study with a 2 × 2 factorial design. Lancet. 2022;399(10336):1695–707. 35405085 10.1016/S0140-6736(22)00367-1

[6] Gudenkauf LM, Gray S, Gonzalez DB, et al. Balancing hormone therapy: mitigating adverse effects of androgen-deprivation therapy and exploring alternatives in prostate cancer management. Am Soc Clin Oncol Educ Book. 2024;44:e433126. 38788186 10.1200/EDBK_433126

[7] Sathekge M, Bruchertseifer F, Vorster M, Lawal IO, Mokoala K, Reed J, et al. 225Ac-PSMA-617 radioligand therapy of de novo metastatic hormone-sensitive prostate carcinoma (mHSPC): preliminary clinical findings. Eur J Nucl Med Mol Imaging. 2023;50(7):2210–8. 36864360 10.1007/s00259-023-06165-9 PMC10199874

[8] Kratochwil C, Fendler WP, Eiber M, et al. Joint EANM/SNMMI procedure guideline for the use of ^177^Lu-labeled PSMA-targeted radioligand-therapy (^177^Lu-PSMA-RLT). Eur J Nucl Med Mol Imaging. 2023;50(9):2830–45. 37246997 10.1007/s00259-023-06255-8 PMC10317889

[9] Steinhelfer L, Lunger L, Cala L, et al. Long-term nephrotoxicity of ^177^Lu-PSMA radioligand therapy. J Nucl Med. 2024;65(1):79–84. 37857504 10.2967/jnumed.123.265986

[10] Borque-Fernando Á, Alonso-Gordoa T, Juan-Fita MJ, Lopez Campos F, Pérez-Fentes DA, Vilaseca A, et al. Beyond the status quo: when disease volume and metastatic timing are not enough to personalize treatment in mHSPC. Future Oncol. 2025;21(8):991–1003. 40029138 10.1080/14796694.2025.2468569 PMC11938960

[11] Ahmadzadehfar H, Rahbar K, Baum RP, et al. Prior therapies as prognostic factors of overall survival in metastatic castration-resistant prostate cancer patients treated with [^177^Lu]Lu-PSMA-617. A WARMTH multicenter study (the 617 trial). Eur J Nucl Med Mol Imaging. 2021;48(1):113–22. 32383093 10.1007/s00259-020-04797-9 PMC7835179

[12] Mirzaei S, Schweighofer-Zwink G, Ofner H, et al. Use of 177Lu prostate-specific membrane antigen therapy in metastatic castration-resistant prostate cancer. Wien Klin Wochenschr. 2025;137(Suppl 4):157–66. 40464929 10.1007/s00508-025-02544-4 PMC12137475

[13] Hakozaki Y, Yamada Y, Takeshima Y, et al. Low hemoglobin and PSA kinetics are prognostic factors of overall survival in metastatic castration-resistant prostate cancer patients. Sci Rep. 2023;13(1):2672. 36792713 10.1038/s41598-023-29634-5 PMC9931698

